# Assessing mobile technology use and mHealth acceptance among HIV-positive men who have sex with men and transgender women in Malaysia

**DOI:** 10.1371/journal.pone.0248705

**Published:** 2021-03-23

**Authors:** Archana Krishnan, Damian Weikum, Claire Cravero, Adeeba Kamarulzaman, Frederick L. Altice

**Affiliations:** 1 Department of Communication, University at Albany, State University of New York, Albany, New York, United States of America; 2 Yale School of Public Health, New Haven, Connecticut, United States of America; 3 Harvard T.H. Chan School of Public Health, Boston, Massachusetts, United States of America; 4 Centre of Excellence for Research in AIDS (CERiA), Faculty of Medicine, University of Malaya, Kuala Lumpur, Malaysia; 5 AIDS Program, Section of Infectious Diseases, Yale School of Medicine, New Haven, Connecticut, United States of America; Helen Keller International, SIERRA LEONE

## Abstract

**Background:**

Mobile health (mHealth) can be beneficial in monitoring the complex healthcare regimen for people with HIV that includes adhering to medication and refraining from risky practices such as unsafe sex and injection drug use. Not only is mHealth often implemented without appropriate feasibility and acceptability research, but there is limited mHealth research among key HIV-positive populations such as men who have sex with men (MSM) and transgender women (TGW).

**Methods:**

This study assessed access to and use of mobile technology and acceptability of mHealth among 150 HIV-positive MSM and TGW who were prescribed antiretroviral therapy (ART) in Malaysia–an emerging economy with rapid telecommunications growth and societal stigma against these groups.

**Results:**

Findings among the 114 MSM and 36 TGW reveal high levels of depression (42%), stigma (2.53/4.00) and risky sexual behavior (30%), and suboptimal ART adherence (22%). On the other hand, the sample had excellent access to smartphones (75.3%) and the internet (78%), and had high acceptance of mHealth especially for those with suboptimal ART adherence.

**Conclusion:**

In settings like Malaysia where homosexuality and cross-dressing are socially and legally stigmatized, HIV prevention and treatment strategies delivered using an mHealth platform have the potential to overcome in-person barriers.

## Introduction

Mobile health (mHealth) is a field of research that centers around the use of mobile devices to manage healthcare, improve health outcomes, and promote preventive health behaviors [1–3]. In response to international guidelines that have recommended scaling back HIV adherence interventions in terms of cost and personnel [4], mHealth has been offered as a complement to existing strategies [5]. mHealth interventions have already shown efficacious results in improving health outcomes related to multiple chronic conditions [6–8], and they can be particularly beneficial in managing the complexity of care for people with HIV (PWH). Compared to other health conditions, HIV care is especially burdensome in terms of cost and effort. PWH need to engage in affirmative activities such as adhering daily to keep their viral load at an undetectable level, and refrain from risky injection drug use and sexual behaviors to prevent transmission [9, 10]. There are however, limited feasibility and acceptability studies of mHealth in general, and limited mHealth research among highly vulnerable groups of PWH such as people who use drugs (PWID), men who have sex with men (MSM) and sex workers [5, 11]. Moreover, there is insufficient evidence to inform the implementation and scale-up of mHealth in various disparate HIV populations.

In many countries, the HIV epidemic is concentrated in specific groups; this is particularly true in a low-to-middle-income country (LMIC) such as Malaysia where MSM and transgender women (TGW) have HIV prevalence 12–18 times than that of the general population [12]. While HIV prevalence in the general Malaysian population is low at around 0.4%, prevalence among key groups is disproportionately high, e.g., 8.9% among MSM and 5.6% among TGW [12]. Multiple reasons–high levels of risk behavior, low levels of knowledge about transmission risk, and low rates of HIV testing among MSM–have been suggested for the increase in HIV incidence [13, 14]. MSM also face substantial societal stigma and legal barriers in Malaysia, a trend that is mirrored in other LMICs in the region such as Indonesia and Cambodia [15]. Specifically, section 377 of the Malaysian Penal Code criminalizes same-sex intercourse and levies imprisonment of up to 20 years. Homosexuality and cross-dressing are also illegal in Shariah law in Malaysia. Recent studies have found high levels of stigma against HIV-positive MSM even amongst medical students and healthcare providers [16, 17]. As a result, MSM and TGW with HIV are amongst the most vulnerable and underserved PWH in Malaysia and often unable to benefit from access to government-subsidized ART.

Malaysia is a fair representation of LMICs both in the Southeast Asian region and globally, with respect to telecommunication infrastructure and consumer base. The adoption of telecommunications in Malaysia, like in the rest of Southeast Asia, has been rapid. Recent reports indicate that Malaysia has a mobile phone penetration rate of 97.5% and Internet penetration of 71.1% [18]. The growth of communication technology in Malaysia, thus holds promise in being able to incorporate mHealth strategies and tools in abetting health management and influencing behavior change. Moreover, there is precedence in the literature that indicates that lesbian, gay, bisexual transgender and queer (LGBTQ) individuals are early adopters of information and communication technologies [19, 20], and hence might benefit from mHealth-enabled interventions. There is however, no published research on the use of mobile technologies and acceptance of mHealth among vulnerable HIV populations in Asia. An ideal opportunity exists in Malaysia to conduct mHealth feasibility work. In response to criticism that mHealth is often implemented in the face of a lack of acceptability work [5, 21] and in recognizing the importance of examining mHealth among vulnerable populations [11], the purpose of this study is to ascertain at a micro level the following: (1) mobile technology access and use, (2) mHealth intervention acceptability, and (3) social and health constructs including drug/alcohol use, mental health and stigma, among HIV-positive MSM and TGW in Kuala Lumpur, Malaysia. The findings will allow us to assess opportunities to implement future mHealth interventions in Malaysia to improve HIV outcomes.

## Methodology

### Study sample and setting

A purposive sample of 150 HIV-positive MSM and TGW was recruited over 12 weeks from the Pusat Perubatan Universiti Malaya clinic and through community outreach in Kuala Lumpur, Malaysia. The Pusat Perubatan clinic is an academic health clinic affiliated with the University of Malaya which provides medical care to the general public and to key populations such as PWH and LGBTQ. A random sample in this context was deemed to be logistically impractical as MSM and TGW are hard-to-reach populations due to considerable social stigma surrounding sexual minorities and HIV in Malaysia. Thus, a convenience approach was utilized [22] by approaching patients at the university clinic and using a research assistant familiar with the MSM and TGW population in Kuala Lumpur. Recruitment was limited to individuals who: 1) self-identified as MSM or TGW, 2) were 18 years or older, 3) were HIV-positive, 4) had been receiving HIV care for at least 12 months prior to study enrollment, and 5) had been prescribed ART 6 or more months prior to study enrollment.

### Procedure

Recruitment included clinic-based advertisements and flyers or clinical staff approaching patients at the end of their clinical visits. Community outreach recruitment occurred through distribution of advertisements and fliers at adherence support meetings and online advertisement on Facebook. After participants were informed of the study and provided written informed consent, they completed a 60-minute computer-assisted survey interview (CASI) in a private setting and were paid 30 Malaysian Ringgit (∼$8 US) for their time. The survey was developed and hosted on the Qualtrics online survey platform, and was administered by a research assistant on a tablet computer. The study protocol was approved by Yale University Human Investigation Committee and the University of Malaya Medical Ethics Committee (protocol # 1404013880).

### Measures

#### Health variables

*Depression* was measured using the 10-item Center for Epidemiologic Studies Depression Scale (CES-D 10) [23] with a cut-off of > 7 to define moderate-to-severe depression. *Stress* was assessed on a 5-point Likert scale using the 4-item perceived stress scale [24]. *Health literacy* was assessed using a 3-item screening instrument measured on a 5-point Likert scale [25]. *Severity of drug use* was measured using the DAST-10 which is a 10-item aggregate score of drug use behaviors. *Severity of alcohol use* specifically was scored using the World Health Organization’s validated 10-item Alcohol Use Disorders Identification Test (AUDIT) [26]. The aggregate score of 40 was categorized with standard cut-offs to indicate severity of alcohol use, specifically normal drinking (0–7), hazardous drinking (8–15), harmful drinking (16–19) and alcohol dependence (≥ 20). *Risky sexual behavior* was measured using two items assessing the following behaviors–unprotected anal intercourse during the last sexual encounter and frequency of unprotected sex in the past three months. *ART adherence* was measured using two items, one which was assessed on a 5-point Likert scale (“How would you rate your ability to take your HIV medications every day over the last month”), and the other assessed on a 0–100% scale (“In the past month, please indicate the percentage of ART medications that you took.”). The latter item was transformed into two binary variables–optimal adherence (≥ 90%) and perfect adherence (100%) based on prior studies [20, 27, 28].

#### Social variables

*Social support* was measured on a 5-point Likert scale using Sherbourne and Stewart’s 19-item social support instrument [29]. Since the population in the present study was both HIV-positive and a sexual minority, two types of stigma were assessed–*HIV stigma* and *sexual orientation stigma*. HIV stigma was measured using the Wright et al. (2007)’s 10-item scale [30] which is assessed on a 4-point Likert scale. Sexual orientation stigma was adapted from Wright et al. [30] by rewording the items to reflect sexual orientation instead of HIV.

#### Technology variables

Self-created instruments were used to measure *mobile phone access* measured categorically and as an open-ended item. *Frequency of mobile phone use* was measured using 14 items on a 5-point Likert scale (e.g., “How often do you engage in sending or receiving text messages on your mobile phone?”). *Frequency of internet use* was measured using 7 items on a 5-point Likert scale. The items were all specific to internet use for general and HIV-related behaviors (e.g., “Indicate how often you search the web for HIV-related health information.”). The 6-item *mHealth acceptance* scale was adapted from Krishnan et al. [20] that were measured on a 5-point Likert scale (e.g., “How likely are you to use mHealth in reminding you to take your HIV medication?”). Mean scores were created for the above scales, and reliabilities ranged from good to excellent (see Table 3).

## Results

The sample was composed of 76% MSM (*n* = 114) and 24% TGW (*n* = 36), and was relatively young (*M* = 35.1 years, *SD* = 8.3), well-educated (58.6% college-educated), and employed (82% full-time). 63.3% identified as homosexual, 15.3% as bisexual, and 20.7% as heterosexual. Table 1 shows the number of participants who met the cut-off for moderate to severe depression, unprotected anal sex, risky sexual behavior, and optimal and perfect ART adherence.

**Table 1:** Medication adherence and HIV risk factors for study participants (N = 150).

Variable	N (%)
Currently taking ART	125 (83.3%)
Optimal ART > = 90%	117 (78%)
Perfect ART = 100%	95 (63.3%)
Unprotected sex (last time)	37 (27.8%)
Unprotected sex (past 3 months)	45 (30%)
Alcohol use disorder (AUDIT)	10 (6.7%)^a^
Substantial drug use (DAST-10)	16 (10.7%)^b^
Moderate-to-severe depression	63 (42%)

^a^ AUD rate of the entire sample. When considering only the number of participants who admitted to drinking alcohol in the last 12 months (*n* = 52), those with AUD is 19.2%.

^b^ None showed severe drug use.

Despite study enrollment being restricted to participants who had been prescribed ART in the past 6 months, only 83.3% were currently taking ART. Although severity of alcohol (6.7%) and drug use (10.7%) were low in this sample, sexual risk was high with a third reporting unprotected sex during the last encounter (27.8%) and in the past six months (30%). Stress, depression and HIV stigma were also relatively high with 42% meeting criteria for moderate to severe depression. On the other hand, the sample had high health literacy (*M* = 4.29, *SD* = 0.85) and high levels of social support (*M* = 3.43, *SD* = 0.89). Table 2 depicts the mean scores for the social and mental health variables.

**Table 2:** Mean scores for social and mental health variables (N = 150).

Variable	Mean (SD)
Perceived stress	2.79/5.00 (0.70)
Social support	3.43/5.00 (0.89)
Health literacy	4.29/5.00 (0.85)
HIV stigma	2.53/4.00 (0.63)
Sexual orientation stigma (n = 114)	2.13/4.00 (0.66)

With respect to mobile technology access, 75.3% had access to smartphones, 28% had access to a tablet, and 20.7% to a standard mobile phone without internet access. Even though only 78% had access to the internet, more than three-fourths accessed it on their mobile devices. Table 3 shows the overall scale and individual item mean scores for frequency of mobile phone use, frequency of internet use, and mHealth acceptance. As can be noted from the table, the most frequent uses of mobile phones were making or receiving phone calls (*M* = 4.22, *SD* = 0.76), and sending or receiving text messages (*M* = 3.99, *SD* = 0.97). With respect to internet use, the most frequent use was searching for general information (*M* = 3.44, *SD* = 1.01) followed by HIV-specific health information searching (*M* = 2.97, *SD* = 1.12). Overall, acceptance of mHealth was higher than average (*M* = 2.61, *SD* = 1.00), with the highest acceptability being towards using mHealth for receiving information about HIV health (*M* = 2.95, *SD* = 1.34) and sexual health (*M* = 2.93, *SD* = 1.32), and for receiving reminders to take HIV medications (*M* = 2.88, *SD* = 1.65).

**Table 3:** Means, standard deviations, and reliabilities of mobile technology use, internet use and mHealth acceptance scales.

Scale/Item	N	Mean	SD	α
**Mobile Phone Use**	**122–138**^**a**^	**3.08**	**0.85**	**.90**
1. Make or receive phone calls	138	4.22	.764	
2. Take a picture	130	3.62	1.044	
3. Record a video	129	2.64	1.09	
4. Send or receive text messages	137	3.99	.966	
5. Access the internet	125	3.30	1.45	
6. Send or receive emails	124	2.76	1.49	
7. Download applications	126	3.04	1.28	
8. Listen to music	133	3.44	1.22	
9. Watch videos	127	3.08	1.25	
10. Online banking	125	2.27	1.42	
11. Play games	129	2.40	1.38	
12. Social media	126	3.61	1.31	
13. Use health-related apps	125	2.18	1.19	
14. Read e-books	122	1.79	1.13	
**Internet Use**	**117**^**b**^	**2.76**	**0.83**	**.87**
1. Search for general information	117	3.44	1.01	
2. Search for health-related information	117	2.97	1.12	
3. Search for HIV-related health information	117	2.91	1.07	
4. Read blogs about HIV experiences, treatment	117	2.73	1.12	
5. Participate in online social support groups for HIV-infected individuals	117	2.07	1.26	
6. Read HIV-related news articles	117	2.73	1.09	
7. Search online for sexual partners	117	2.45	.98	
**mHealth Acceptance**	**150**	**2.61**	**1.00**	**.84**
1. Assess your health behaviors	150	2.58	1.24	
2. Remind you to take your HIV medications	150	2.88	1.65	
3. Assess alcohol/drug behaviors	150	1.92	1.18	
4. Assess sexual behaviors	150	2.38	1.27	
5. Receive HIV-related health information	150	2.95	1.34	
6. Receive information about sexual health	150	2.93	1.32	

^a^ Response limited to those who had access to a mobile phone; furthermore, some participants did not respond to specific items (e.g., if their mobile phone did not have a camera feature).

^b^ Response limited to those who had access to the Internet.

To gauge if mental health status of participants acted as a barrier to mobile technology use and mHealth acceptance, multiple regressions were conducted. A significant equation was found predicting mobile phone use from stress and depression at *F* (2, 136) = 4.70, *p* < .05, R^2^ = .07; however, only stress was a significant predictor at *t* = 3.03, *p* < .01, *β* = .25. This significant result shows that stress was not a barrier and in fact was positively associated with frequency of mobile phone use. Significant equations were not found for stress and depression predicting internet use and mHealth acceptance at *F* (2, 114) = 1.74, *p* = .18 and *F* (2, 147) = .12, *p* = .89 respectively. This indicates that mental health was not a barrier to either internet use or mHealth acceptance.

Suboptimal ART adherence is associated with poor HIV treatment outcomes [31, 32] and is thus a prime outcome for behavioral change interventions. Thus, it is important to assess if acceptability of mHealth varies among those who are optimally adherent or not, and those who are perfectly adherent or not. A series of independent samples t-tests (see Table 4) was conducted to look for significant differences in mHealth acceptance among those who had optimal adherence and perfect adherence. A statistically significant difference was found between those who reported being optimally adherent and those who did not. i.e., those who were not optimally adherent reported higher acceptability of mHealth. No significant difference was found between groups with and without perfect adherence.

**Table 4:** Differences in mHealth acceptance among optimal and perfect adherence groups.

	Optimal Adherence					
	> = 90%		< 90%			
	Mean	SD	Mean	SD	df	t
mHealth acceptance	2.56	.99	3.27	1.13	123	1.98*
	Perfect Adherence					
	= 100%		< 100%			
	Mean	SD	Mean	SD	df	t
mHealth acceptance	2.51	1.01	2.88	.95	123	1.76

*p < .05.

## Discussion

Our findings provide a micro-level perspective of both the public health and technology make-up of a sample of HIV-positive MSM and TGW living in Malaysia. The sample had high levels of depression and risky sexual behavior which unfortunately is typical in a socially- and medically-underserved HIV population. First-line HIV medications are fully government-subsidized for all Malaysian nationals. However, despite being eligible to receive HIV medication and study enrollment being restricted to participants who had been prescribed ART in the past 6 months, almost a fifth of the sample was not on an ART regimen. This is an indirect measure of healthcare stigma against HIV-positive MSM and TGW, one that is corroborated by high perceived stigma scores in this study and one that has been highlighted by previous research. Earnshaw et al. [16] and Ferro et al. [17] showed high levels of discrimination against PWH in Malaysia by healthcare providers and the detrimental effect it had on treatment intention, especially if PWH were a sexual minority or had co-occurring issues such as alcohol and drug use disorders. Suboptimal levels of ART access among vulnerable populations such as MSM and TGW have been documented elsewhere [27, 28, 33, 34].

Among the participants who were on ART, one-fourth were not optimally adherent to their medication regimen. More troubling, a third reported high-risk sexual behaviors. This reveals the extreme vulnerability of this group of PWH along with a treatment gap that needs to be addressed. Suboptimal ART adherence is associated with incomplete viral suppression which increases HIV transmission risk by those who continue to engage in high-risk sexual behaviors. Achieving optimal ART adherence, as part of the treatment and prevention approach, is thus crucial to reduce HIV transmission and improve survival [35, 36]. This is precisely why most interventions for PWH tend to focus on improving ART adherence. In fact, a meta-analysis found that those who participated in adherence-enhancing interventions were significantly more likely to achieve 95% adherence and viral load suppression [37].

As international guidelines suggest scaling back HIV interventions in terms of cost and personnel [4, 11], mHealth is poised to fulfill this need. This study reveals an opportunity to implement mHealth–a majority of the sample had access to smartphones. In fact, mobile phone ownership rates were higher than that of the general population in Malaysia [18]. Similarly, a majority were accepting of mHealth to assess their drug use and improve their medication adherence among others. More importantly, not only was mental health status not a barrier to using mobile technology and the internet, but mHealth acceptance was higher among those with suboptimal adherence. mHealth can be particularly advantageous in resource-limited settings [7, 38], thus making its use particularly appealing in an emerging economy such as Malaysia. Moreover, the sample had high health literacy and high levels of social support, both of which can add valuable support to adherence interventions.

This study has shown strong preliminary evidence of both a gap and an opportunity that exists in Malaysia, to implement mHealth among underserved PWH. There has not any previous examination in the region that lays out the feasibility of implementing an mHealth intervention among underserved PWH. The findings in this study echo previous findings that showed high acceptability of mHealth among PWH, albeit in other geographical regions [20, 39]. There is strong evidence for an mHealth-based intervention among HIV-positive MSM and TGW who are not optimally adherent to ART. The focus of future mHealth interventions must be both on decreasing risky sexual behavior and improving ART adherence, both of which can be monitored in real-time or near real-time and influenced by disseminating tailored health communication messages. Furthermore, since low rates of HIV testing are partially to blame for rising rates among MSM, ubiquitous mobile phone use can be leveraged to disseminate information about HIV testing.

Key HIV populations like MSM and TGW are not only underserved with respect to medical access and care, but efforts to keep them engaged with medication regimens are fraught with stigma and discrimination sometimes by the very people treating them. mHealth interventions can to a certain extent, provide support and protection against discriminatory behavior. Moreover, high levels of technology use and mHealth acceptance among HIV-positive MSM and TGW in Malaysia provide strong evidence for developing and implementing a technology-based adherence intervention. This is likely to address the urgent need for innovative yet cost-effective strategies to address treatment gaps for underserved HIV populations.
